# CAMHS Emergency Assessment Service (EAS): development & implementation during the COVID-19 crisis

**DOI:** 10.1192/bjo.2021.193

**Published:** 2021-06-18

**Authors:** Tania Saour, Jovanka Tolmac, Braulio Girelas, William Turton, Lauren Branney

**Affiliations:** Central North West London NHS Trust

## Abstract

**Aims:**

- To provide emergency psychiatric assessment throughout the COVID-19 pandemic.
- To maintain patient and staff safety by minimising exposure to infection risk by reducing A&E contact.
- To alleviate pressures on the A&E department by enabling CAMHS patients be seen in an alternative setting.
- To provide a more appropriate environment for the assessment of young people in acute distress.

**Method:**

- Service live 8th April 2020 to 8th June 2020.
- Exclusion criteria: 1) confirmed/suspected overdose; 2) self-harm with injuries requiring medical attention; 3) acute psychotic episode; 4) drug/alcohol intoxication; 5) high risk of absconding (ASD/LD/LAC), 6) severe agitation/aggression; 7) eating disorders requiring medical intervention; 8) section 136 of the MHA; 9) break down of a social care placement; 10)medically unexplained symptoms.

Data reviewed of all young people who were referred to A&E during March–April 2020. Each case was assessed as to whether they were then seen within the EAS Service.

These cases were reviewed demographically looking at ethnicity, gender, while also reviewing the reason for referral.

**Result:**

- A total of 90 cases referred to Urgent Care Team
- Nineteen (21%) met criteria for assessment at EAS
- 80% of presentations between 12am and 9am.
- Commonest reasons for referral : low mood with suicidal ideation (42%), anxiety (26%)
  - 50% service users not previously known to CAMHS
- Majority of service users were female
- Mean age 15 years
- All but one of the young people assessed at the EAS, were discharged home with community follow-up

**Conclusion:**

- Average total no. monthly referrals to CAMHS Urgent Care Team (UCT) fell from approx. 90 to 45.
- Only a small proportion of referrals (21%) could be safely seen by the EAS, suggesting that the majority of young people required a joint assessment by A&E and CAMHS Urgent Care Team.
- When need arises, very rapid reconfiguration and implementation of CAMHS emergency services is achievable.
- EAS diverted a small number of young people from exposure to COVID-19 in A & E.
- The service was set up speedily without evaluation of parent/carer/young people views or evaluation of cost-effectiveness.
- If similar services are to be set up permanently, the balance between safety and the risk of division between mental & physical health services and potential to increase stigmatisation of mental illness should be considered.
- Adaptation to future outbreaks should be informed by this initiative.

